# Tracking Molecular Recognition at the Atomic Level with a New Protein Scaffold Based on the OB-Fold

**DOI:** 10.1371/journal.pone.0086050

**Published:** 2014-01-20

**Authors:** John D. Steemson, Matthias Baake, Jasna Rakonjac, Vickery L. Arcus, Mark T. Liddament

**Affiliations:** 1 Department of Biological Sciences, University of Waikato, Hamilton, New Zealand; 2 Institute of Molecular BioSciences, Massey University, Palmerston North, New Zealand; 3 OBodies Limited, Hamilton, New Zealand; Technical University of Braunschweig, Germany

## Abstract

The OB-fold is a small, versatile single-domain protein binding module that occurs in all forms of life, where it binds protein, carbohydrate, nucleic acid and small-molecule ligands. We have exploited this natural plasticity to engineer a new class of non-immunoglobulin alternatives to antibodies with unique structural and biophysical characteristics. We present here the engineering of the OB-fold anticodon recognition domain from aspartyl tRNA synthetase taken from the thermophile *Pyrobaculum aerophilum*. For this single-domain scaffold we have coined the term OBody. Starting from a naïve combinatorial library, we engineered an OBody with 3 nM affinity for hen egg-white lysozyme, by optimising the affinity of a naïve OBody 11,700-fold over several affinity maturation steps, using phage display. At each maturation step a crystal structure of the engineered OBody in complex with hen egg-white lysozyme was determined, showing binding elements in atomic detail. These structures have given us an unprecedented insight into the directed evolution of affinity for a single antigen on the molecular scale. The engineered OBodies retain the high thermal stability of the parental OB-fold despite mutation of up to 22% of their residues. They can be expressed in soluble form and also purified from bacteria at high yields. They also lack disulfide bonds. These data demonstrate the potential of OBodies as a new scaffold for the engineering of specific binding reagents and provide a platform for further development of future OBody-based applications.

## Introduction

Molecular recognition is a crucial aspect of a successful biological system. High-affinity interactions govern immune function, while relatively transient events are seen in signal transduction. Each type of interaction is dependent on the adaptability of protein folds during evolution. We have explored this phenomenon using a widely-distributed protein domain, the OB-fold, originally named for observed functions of oligosaccharide and oligonucleotide binding [1].

The OB-fold is a 5-stranded β-barrel domain that presents a concave binding face. In virtually all cases where the domain is present, this same face is used for binding ligand. A survey of the SCOP database reveals many OB-folds which are heavily modified with additional loops or entirely new domains inserted [2]. Examples of OB-folds can be found in diverse organisms, including archaea, bacteria, yeast and mammals, with no detectable sequence conservation across the superfamilies and representing a diverse range of natural ligands, including proteins, oligonucleotides, oligosaccharides and small molecules. These combined factors led Murzin to suggest that the OB-fold is ancient and tolerant to mutation, with an easily-adaptable binding face [1]. Ultimately the unifying feature of the OB-fold is not sequence, but structure and topology, with architecture capable of supporting a very wide range of sequences and modifications [3]. These observations suggested that the plasticity of the OB-fold might be exploited *in vitro* through protein engineering for specific molecular recognition.

Antibodies and antibody fragments are currently the dominant class of engineered proteins for molecular recognition; very large combinatorial libraries (in excess of 10^11^ individual members) have been made and selected *in vitro* to obtain binding reagents with very high affinity and specificity (reviewed in [4]–[6]). However, some general limitations of the antibody format exist, such as their large size, requirement for glycosylation (in the case of full-length antibodies) and a critical dependence on intrachain disulfide bonds, a limitation shared by even the smallest fragments of antibodies (individual V domains, termed domain antibodies [7]). Driven by a desire to circumvent these limitations, a need to expand the useful target range that can be addressed pharmacologically and also to avoid potentially limiting issues of intellectual property, the field of non-antibody protein scaffolds has developed rapidly over the last decade (recently reviewed in [8]–[10]). Affibodies (based on a three-helix-bundle Z domain from staphylococcal protein A), have achieved high affinities against a range of protein targets [11]–[13]. Anticalins are high-affinity small-molecule binders that were selected from libraries of a mutant lipocalin fold [14]. Designed Ankyrin repeat proteins (DARPins) have been engineered with sub-nanomolar affinities for protein targets [15]. The Sac7d DNA-binding protein, an SH3-like five-stranded incomplete β-barrel (mistakenly identified as an OB-fold [16]) from the hyperthermophile *Sulfolobus acidocaldarius* has successfully been used as a scaffold to produce high-affinity binders to a range of targets – these were initially called Affitins, but later renamed Nanofitins [17]. Engineered derivatives of variable lymphocyte receptors, termed Repebodies, have also recently been described with affinities in the nanomolar range [18]. Given the large number of proteins investigated for engineering purposes, there are relatively few crystal structures of engineered protein-protein complexes. Hogbom et al [19] claimed the first example with an Affibody, and other examples have been published, most notably those of an Anticalin [20], a DARPin [21] and two Repebodies [18].

In principle, OB-folds possess features granting potential benefits to an engineered OB-fold domain over and above antibodies based on the immunoglobulin fold; they are small, single-domain binding modules and generally lack disulphide bonds. Due to their ubiquitous nature, potential OB-fold-based scaffolds may be sourced from widely diverse organisms, including human proteins for therapeutic and *in vivo* diagnostic use, or thermophilic microorganisms for more general applications. Given that OB-folds have a unique antigen binding site (formed from a combination of concave β-sheet and loops), it may be possible to favour different binding solutions than those seen in scaffolds where loops are the principal mediators of specificity and affinity, such as immunoglobulins.

In this study we have designed and tested the properties of a new protein scaffold based on the OB-fold of the aspartyl tRNA synthetase (AspRS) from the thermophile *Pyrobaculum aerophilum.* We term these small, single-domain engineered binding proteins OBodies. To demonstrate the amenability of this new scaffold to engineering for new binding specificity, we developed a series of OBodies with increasing affinity to a model target protein, hen egg-white lysozyme (HEL). Parallel crystal structures of OBody:HEL complexes have given us a molecular-level view of the affinity maturation process, as we follow binders from micromolar to nanomolar affinity. Sharing a common range of epitopes centred on the HEL active site, these OBodies were also found to be efficient inhibitors of HEL activity. These data demonstrate the potential of OBodies as a new scaffold and suggest that a broad new class of binding reagents based on other OB-folds may be feasible.

## Results

### Design, Construction, Selection and Validation of a Naïve Library Based on the OB-fold from the Pyrobaculum Aerophilum Aspartyl tRNA Synthetase

**Figure 1:**
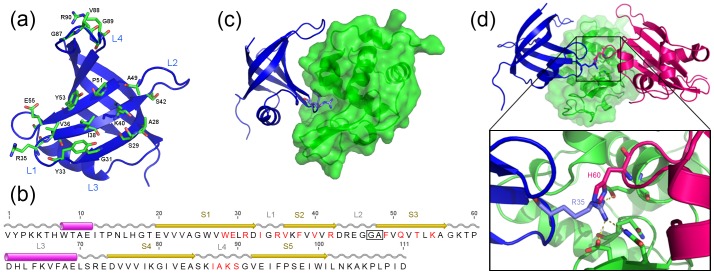
Naïve library design and NL8 structure. (a) Model of *Pyrobaculum aerophilum* AspRS, derived from the crystal structure of NL8 and showing the naïve library design. The structure is displayed in blue cartoon representation, with the 17 residues randomised shown as green stick models. (b) Schematic of naïve library design. Residues randomised in the library by NNK codons are shown in red. The annotated secondary structural elements, derived from the crystal structure of NL8, are shown above the sequence as yellow arrows (β-strands; S1–5), pink cylinders (α-helices) and grey wavy lines (loops; L1–4). The GA residues (positions 46 and 47; boxed) are the result of a 6 bp insertion in the wild-type gene immediately following wild-type residue 46, done to facilitate library construction. (c) The NL8 OBody (blue) is shown in complex with HEL (green surface model). Highlighted as stick models are R35 and Y33, interacting with the substrate-binding groove of HEL. (d) The R35 of the NL8 Obody (blue) binds into the HEL active site, in a similar manner to H60 from lysozyme inhibitor protein YkfE from *E. coli* (pink).

To generate a naïve library, 17 specific sites on the surface of the domain were selected based on surface accessibility and their interaction with the native tRNA substrate, using a homology model built by the Swiss Model automated server [22]. These residues were randomised by PCR using multiple overlapping oligonucleotides containing degenerate NNK codons (Figure 1a and b) in phagemid pRpsp2 [23], generating a library comprising 10^8^ independent clones. To facilitate library construction using this strategy, 6 bp (encoding the residues GA) were inserted into loop 2 of the wild-type AspRS OB-fold gene immediately following residue 46 to provide adequate annealing overlap for the mutagenic oligonucleotides used in that region (Figure 1b, Figure S4). It was reasoned that an insertion here would be well-tolerated and unlikely to destabilise the AspRS OB-fold domain, an assumption that was proven correct by the subsequent performance of the library in selections and also crystal structures described below.

Initial solid-phase phage display selections were done over five rounds, using immobilised HEL. Monoclonal phage isolates were screened for binding by phage ELISA, and positive clones were expressed in *E. coli*. Surface plasmon resonance (SPR) analysis with immobilised HEL and purified OBodies identified binders of moderate affinity (best NL8, K_D_ = 35 µM; Figure S1). To investigate both the nature of the OBody-HEL complex and provide a structural platform for further development of the domain as a scaffold, the crystal structure of OBody NL8 in complex with HEL was solved to 2.75 Å (Figure 1c and d, Table 1). The structure showed, in atomic detail, a classical protein-protein interaction surface; burial of ∼1600 Å^2^ total solvent-accessible area (823 Å^2^ from the OBody) with a central hydrophobic patch made up of OBody residues Y33, V36 and I38, surrounded by residues making polar contacts (11 hydrogen bonds) and three inter-domain complementary electrostatic interactions. The concave β-sheet binding surface of the OBody wrapped around the HEL target and inserted β-strand one (β1) into the HEL substrate binding groove. The active site of the enzyme was occupied by R35 from the OBody, in a manner similar to H60 from the known HEL inhibitor YkfE [24] (Figure 1d). Interdomain contacts made around the edges of the binding face were made by wild-type residues. In particular, acidic residues D32 and E93 of the OBody mediated electrostatic bonds with complementary basic residues on the bound HEL. The loop 4 (L4) residues of the OBody (positions 87–91) were ordered poorly and did not appear to make any substantive contacts with HEL. However, immediately adjacent to the randomised region, K86 made hydrophobic contacts via the aliphatic carbons on the lysine side chain, and a hydrogen bond via the backbone carbonyl. Computational analysis by Robetta alanine scanning [25] and KFC2 [26] servers predicted the position of hotspots, indicating that binding was focused around Y33, R35 and E93.

**Table 1:** Data collection and refinement statistics for all OBody-HEL complexes reported here.

	NL8	AM1L10	AM2EP06	AM3L15	AM3L09
**Data collection**					
Space Group	p4_1_2_1_2	p2_1_2_1_2_1_	p2_1_2_1_2_1_	p2_1_	p1
Cell dimensions					
a, b, c (Å)	76.759,76.759, 166.344	60.54, 186.26, 245.70	50.43, 58.33, 81.82	51.99, 58.83, 95.18	59.00, 69.38, 76.36
α, β, γ (°)	90	90	90	90, 95.43, 90	72.17, 69.46, 77.55
Wavelength (Å)	0.95666	0.95666	1.54179	1.54179	0.95369
Resolution (Å)	34.9 - 2.69 (2.76 - 2.69)^a^	29.76 - 1.95 (2.05 - 1.95	34.58 - 1.86 (1.96 - 1.86)	35.09 - 1.86 (1.96 - 1.86)	51.50 - 2.57 (2.71 - 2.57)
Measured Reflections	144772	2883973	143392	130001	558386
Unique Reflections	14295 (908)	201772 (27775)	20797 (2819)	46187 (6490)	32670 (4634)
Multiplicity	9	14.3 (12.4)	6.9 (6.5)	2.8 (2.7)	7.8 (7.7)
Completeness (%)	99.15 (92.8)	99 (95.0)	99.4 (94.1)	95.7 (92.3)	96.2 (93.6)
***R***merge (%)†	7.3 (54.3)	6.4 (48.4)	3.9 (22.6)	8.2 (53.3)	11.3 (42.2)
*<I/*σ*(I)>*	32.5 (3.6)	24.5 (5.0)	29.2 (7.5)	8.1 (1.8)	14 (4.9)
Wilson B (Å2)	65	28.19	26.4	22.9	29.7
Mosaicity (°)	0.6	0.2	0.5	0.7	0.7
**Refinement**					
Resolution (Å)	27.5–2.75 (2.82 - 2.75)	29.76- 1.95 (2.01 - 1.95)	34.58 - 1.86 (1.91 - 1.86)	34.58 - 1.86 (1.91 - 1.86)	44.83 - 2.57 (2.67 - 2.57)
*R* factor (%)‡	22.9 (26.5)	18.8 (21.34)	17.18 (20.8)	17.32 (28.10)	20.14 (23.63)
Reflections	12,789 (908)	191,052 (12,500)	19654 (1229)	43800 (3090)	31848 (2736)
*R*_free_ (%)	29.64 (37.6)	22.97 (27.03)	21.5 (29.4)	20.24 (30.8)	24.48 (31.43)
Free reflections	667 (55)	10136 (674)	1061 (57)	2336 (165)	1656 (116)
Refined Atoms	3379	18,959	2101	4381	8155
Protein Chains	4	18	2	4	8
r.m.s.d bond lengths (Å)	0.013	0.007	0.021	0.023	0.004
r.m.s.d bond angles (°)	1.452	1.052	1.854	1.863	0.762
B factor (mean)	51.15	30.20	37.77	26.34	26.8

^a^ Values in parentheses are for highest-resolution shell.

†*R*merge = Σ|*I*_obs_ − <I>|/Σ*I*_obs_.

‡R = Σ||*F*_obs_| − |*F*_calc_||/Σ|*F*_obs_|.

### Affinity Maturation of HEL-binding OBodies: First Round

Examination of the NL8-HEL structure showed a poorly-ordered L4, with electron density visible only for the backbone atoms, suggesting it made little contribution to binding. This loop was therefore targeted for randomisation and also the region randomised was extended by two residues to improve its chances of making contact with HEL.

At the β-sheet face, non-contacting residues S29 and A56 were identified as being small and close to the interface, suggesting that selection of larger residues at these positions may enable additional interactions with HEL. Residues K37 and P51 were identified as making possible negative contributions to binding; P51 because it is a β-breaking residue located on a β-strand, and K37 because of proximity to the guanidinyl group of R61 from HEL, leading to a like-charge clash.

**Figure 2:**
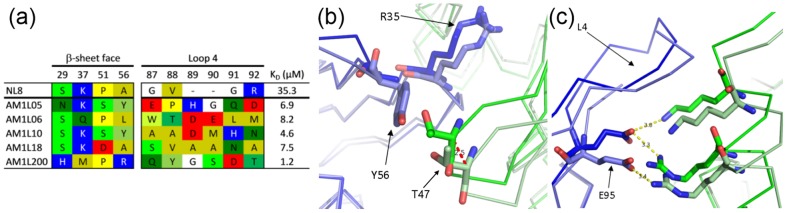
Sequences and affinities of OBodies from first round of affinity maturation and AM1L10 structure. (a) OBody sequences from the first round of affinity maturation, showing only the mutations at the targeted residues and labelled according to their sequence position in the library. For comparison, the equivalent positions from NL8 are also shown. Residues are coloured by polarity or charge (yellows = non-polar, greens = polar, red = acidic, blue = basic). (b) AM1L10 (pale blue Cα trace) in complex with HEL (pale green), superposed on to NL8 (dark blue, r.m.s.d. 0.45 Å) with the NL8-bound HEL also visible (dark green). Substitution A56Y introduces a hydrogen bond with HEL T47 and the subsequent relative shift in HEL position is evident, with a 2.5 Å shift in Cα position at that residue (indicated by red dashed line). (c) AM1L10 (pale blue Cα trace) in complex with HEL (pale green), superposed on to NL8 (dark blue, r.m.s.d. 0.45 Å) with the NL8-bound HEL also visible (dark green). At the top of the binding face, E95 accommodates the shift with a conformational change, maintaining close contact with a lysine and arginine from HEL. Dashed yellow lines are potential hydrogen or electrostatic bonds, labelled with distances in angstroms.

A total of 10 residues in NL8 were therefore randomised (Figure S5) by PCR using multiple overlapping oligonucleotides containing degenerate NNK codons, with the exception of residue 29, which was randomised using an NRK codon. The subset of amino acids encoded by NRK is biased towards bulkier residues, which it was thought would be beneficial at this position. The resulting library in pRpsp2 comprised 10^7^ independent clones. Solid-phase selections using immobilised HEL resulted in fixation of five unique sequences after round six (Figure 2a). All five variants showed improvements in affinity for HEL, ranging from 1–8 µM (determined by SPR; Figure S2). A consensus was clear in the β-sheet face residues, but absent in the L4 residues. The crystal structure of one variant, AM1L10 (K_D_ = 5 µM; Figure 2a), was solved in complex with HEL by molecular replacement to 1.95 Å (Figure 2b and c).

Analysis of the AM1L10 structure gave a clear rationale for the observed improvement in affinity: the substitution A56Y introduced a new hydrogen bond and increased buried surface area. However, analysis by the Robetta [25] virtual alanine scanning server indicated that the predicted energetic contribution to binding by Y56 (ΔΔG = 0.86 kcal/mol) was not sufficient to completely account for the observed change in affinity. Comparison with the NL8-HEL structure showed a moderate rigid-body shift in the arrangement of the OBody-HEL interaction relative to the parent OBody NL8-HEL interface, constituting a rotation centred on R35 of approximately 10° (Figure 2b and c). Residue K37 was re-selected to parental type, but the like-charge clash appeared to be ameliorated by a conformational shift in R61 away from the interface. The L4 residues remained predominantly disordered in the AM1L10 structure (residues D89, M90, H91 and N92 could not be resolved), suggesting that these residues were probably not involved in significant interactions with HEL, with the possible exception of pre-binding long-range electrostatic associations. Surprisingly, substitution P51S did not result in an appreciable structural change, which suggested that any advantage this substitution granted over parent-type may have been a manifestation of different dynamics at that position as well as surrounding residues.

For the next round of affinity maturation, AM1L200 was chosen as the parental clone over AM1L10. This was done for two main reasons: firstly, AM1L10 appeared only once in sequencing of individual phage clones from outputs, compared to multiple instances for all other unique AM1 sequences, suggesting possible problems with AM1L10 display or expression. Secondly, AM1L200 appeared to satisfy better the rational basis for selection of the randomised residues; S29H and A56R introduced large, polar residues and K37M removed the like-charge clash.

### Affinity Maturation of HEL-binding OBodies: Second Round

**Figure 3:**
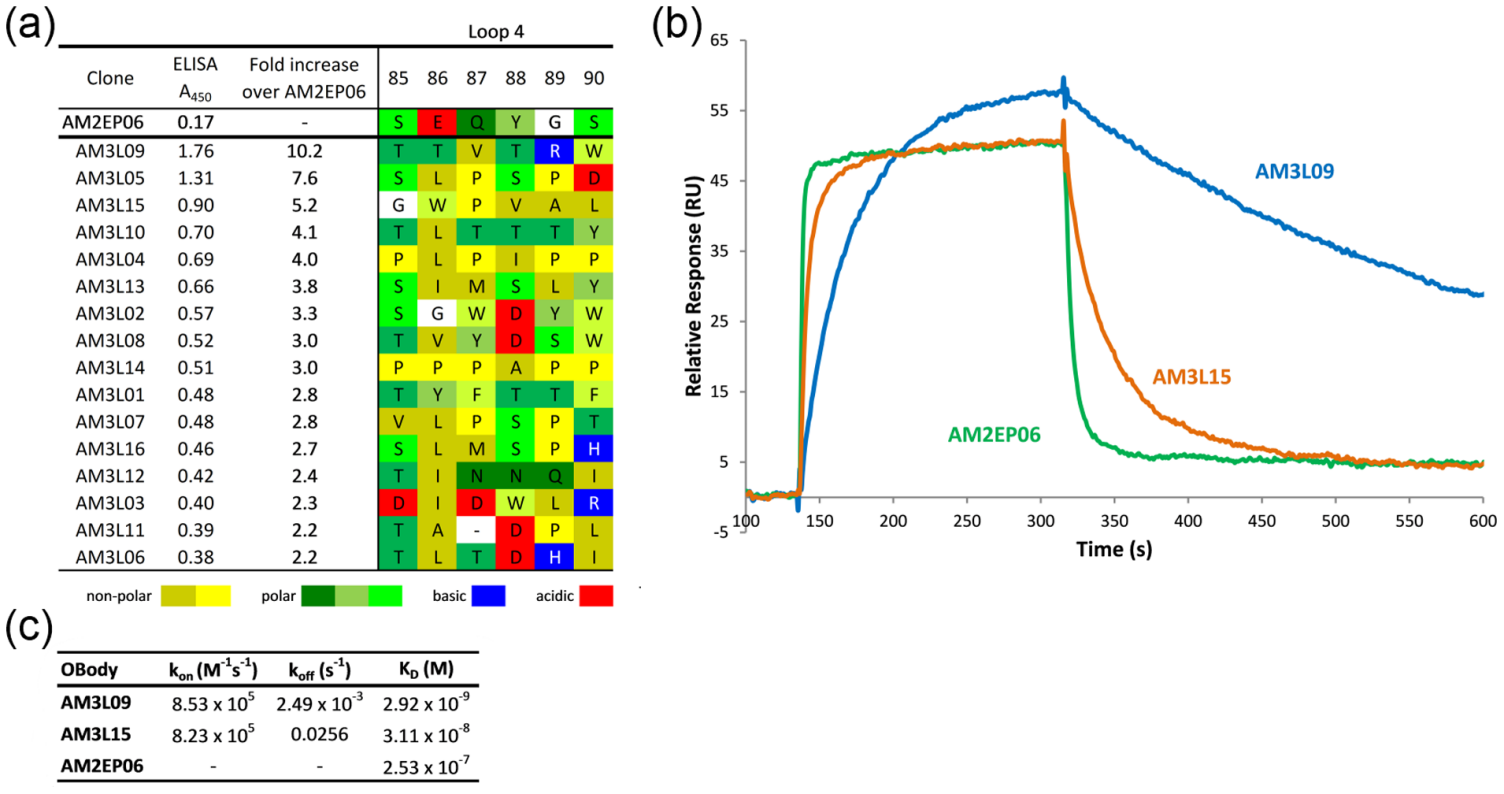
Phage ELISA, SPR and kinetic data for top hits from third round ofaffinity maturation. (a) Top hits from the third round of affinity maturation ranked by phage ELISA signal and showing the corresponding L4 sequence. (b) SPR sensorgrams for OBodies AM3L09 (blue line), AM3L15 (orange line) and AM2EP06 (green line) binding to HEL. For visual comparison, sensorgrams are shown only for the highest single concentration of each OBody analysed (AM3L09 at 32 nM, AM3L15 at 128 nM and AM2EP06 at 800 nM). These data were produced on a single chip and are representative of multiple independent analyses, performed with separate chips and protein samples. (c) Kinetic data for each OBody. The AM3L09 and AM3L15 kinetic data were calculated using Graphpad Prism association/dissociation modelling, whereas the affinity of AM2EP06 was calculated using an equilibrium model of maximum response. While the k_on_ for both AM3L15 and AM3L09 are essentially the same, the difference in dissociation constant can be attributed to a substantial decrease (10-fold) in k_off_. Separate on and off rates could not be determined from the AM2EP06 data.

It was hypothesised that sparse random sampling of a wider sequence space throughout the whole AM1L200 OBody might yield improvements in affinity by introducing beneficial substitutions at sites other than the natural binding face or L4. Accordingly, full-length AM1L200 was subjected to error-prone (EP)-PCR. The resulting library was cloned into phagemid pRpsp2 and comprised 10^8^ independent members; sequencing showed that there were an average of 4 nucleotide substitutions per 1000 bp. Solid-phase selections using immobilised HEL resulted in the isolation of clone AM2EP06, with an improved K_D_ of 250 nM, as determined by SPR (Figure 3b and c).

The crystal structure of AM2EP06 was solved in complex with HEL to a resolution of 1.86 Å. With three amino acid substitutions compared to the parent AM1L200 (T19S, M37K and K86E), it showed a binding face that was very similar to AM1L10 and NL8, with differences at the periphery. In a manner very similar to the AM1L10 structure, a small conformational change in R61 from HEL moved its guanidinyl group away from the interface.

**Figure 4:**
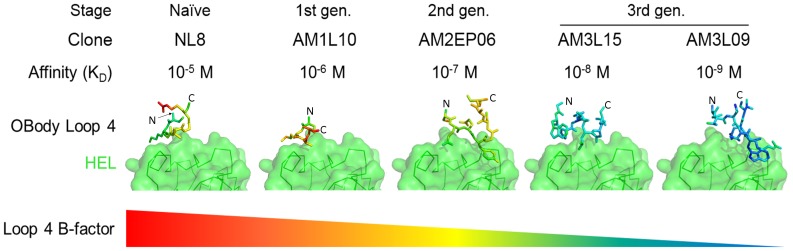
Contribution of L4 to binding in five OBody-HEL complexes. HEL is shown in green as a surface representation in identical orientations in each image and the L4 residues from each OBody variant, coloured according to B-factor relative to the average in each individual case; blue is low B-factor, graduating through green, yellow then red as it increases. The lower-affinity variants NL8 and AM1L10 both show poorly-ordered L4 residues. Note that residues D89, M90, H91 and N92 are missing from the model of AM1L10 L4, as they could not be resolved. Relative stabilisation is evident in L4 of the higher-affinity variants AM3L15 and AM3L09, compared to the parent clone AM2EP06, implying increased involvement in binding. Although the L4 structures of AM3L09 and AM2EP06 have superficially similar configurations, with Y88 and W90 binding in similar positions, W90 L4 from AM3L09 makes a greater number of contacts and is packed more closely. The alpha carbons of N- and C-terminal L4 residues have been labelled with ‘N’ and ‘C’ respectively, to denote the anchor points for the loop in each case.

In contrast to the previous two HEL-complex structures, L4, with an entirely new sequence, was moderately well-defined as electron density in the structure. The loop showed some contact with HEL, increasing buried surface area by ∼100 Å^2^, although no additional hydrogen bonds or other polar contacts were noted. The contribution of L4 to ΔG of binding to HEL appeard to be improving, which was suggested by the crystallographic B-factors. In the previous structures, atoms in the interface had higher B-factors than the average B-factor across the structure, whereas in AM2EP06, while L4 still showed B-factors consistent with a flexible surface loop (well above the average atomic B-factor), B-factors were slightly reduced compared to that seen for L4 in NL8 and AM1L10(Figure 4). The L4 residues from AM2EP06 also made extensive contacts with the β-sheet binding face via S85 and Q87, potentially introducing conformational restraints to HEL-binding residues (Figure S3). Similarly, at the C-terminal end of L4 an intrachain hydrogen bond between S90 and T92 also restrained the loop.

### Affinity Maturation of HEL-binding OBodies: Third Round

Examination of the AM2EP06-HEL complex structure showed that few strong contacts with HEL were made by L4 residues. The third affinity maturation library therefore randomised six residues in L4 of AM2EP06 using a modified version of this sequence as the template, in which a stop codon was introduced by site-directed mutagenesis at position 90 in L4 (AM2EP06-stop). This construct ensured that only clones with a randomised L4 sequence would be functional in the resulting library. The pRpsp2 phagemid was also modified to remove a 2-residue duplication in the pelB leader and revert it to wild-type sequence, creating phagemid pAS1. Residues S85, E86, Q87, Y88, G89 and S90 of AM2EP06 were then randomised using oligonucleotides containing NNS codons by Kunkel mutagenesis [27] in phagemid pAS1, to generate a library comprising 10^9^ independent members. It was estimated by sequencing that L4 was randomised in 75% of clones; the remainder of the library was AM2EP06-stop.

Three rounds of solution-phase selection were done using decreasing concentrations of biotinylated HEL (1 µM, 10 nM and 1 nM across rounds 1–3, respectively). A total of 192 randomly-picked variants were screened by phage ELISA from rounds two and three of selection. Sixteen unique clones were identified with an ELISA signal at least 2-fold higher than the parent clone AM2EP06 (AM3L01-16; Figure 3a). Inspection of the L4 sequences showed a general paucity of charged species, high incidence of proline and convergence towards hydrophobic (L4 residues 86, 87 and 89) or small hydrophilic residues (L4 residue 85). These data suggested a common selective pressure at the randomised positions, but no clear consensus emerged. Based on the ELISA response, two of the top four hits (AM3L09 and AM3L15) were analysed in detail by SPR alongside the parent, AM2EP06 (Figure 3b). The highest affinity attained was 3 nM for variant AM3L09. To examine the structural basis for the observed affinity improvement, this variant along with AM3L15 (K_D_ = 31 nM), was crystallised in complex with HEL and the structures solved to 2.57 Å and 1.86 Å, respectively.

### Anatomy of a nM HEL-binding Obody

**Figure 5:**
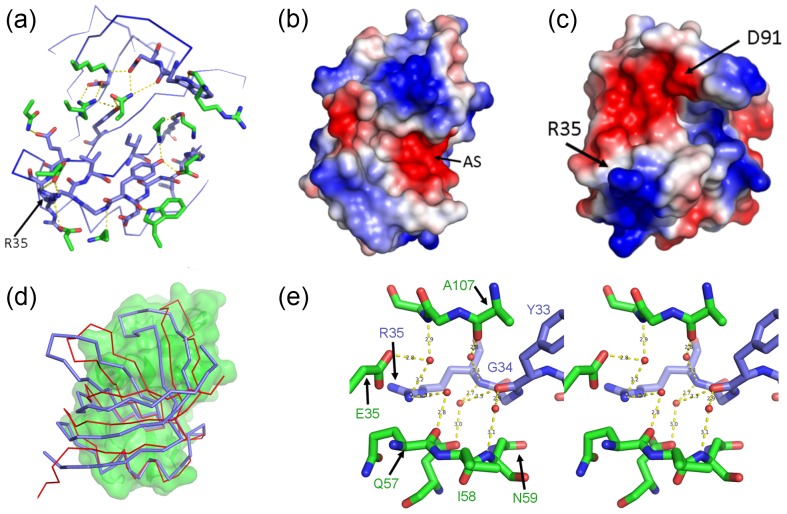
Anatomy of the AM3L09-HEL interface. (a) AM3L09 is coloured blue with interface residues shown as stick models. HEL residues calculated to make a hydrogen bond with AM3L09 are shown as green stick models. Potential hydrogen bonds are indicated by a dashed yellow line. (b) HEL electrostatic surface. The highly electronegative HEL active site (AS) is filled by R35 from AM3L09. (c) AM3L09 electrostatic surface, shown in the same orientation as panel a. The negatively charged patch containing D91 associates with a complementary positively charged patch on the HEL interface. (d) Comparative binding positions of AM3L09 (blue, thick Cα trace) and NL8 (red, narrow Cα trace) to HEL (green surface). (e) The AM3L09-HEL interface, shown in wall-eye stereo. Bridging water molecules between AM3L09 (blue) and HEL (green) are shown as red spheres. Potential hydrogen bonds are indicated by a dashed yellow line labelled with the length in angstroms.

The interface between AM3L09 (K_D_ = 3 nM) and HEL buries a total 1800 Å^2^ solvent-accessible surface area (SASA; 923 Å^2^ from the OBody) and maintains the intimate association with the HEL active site and substrate-binding groove as first established in the NL8-HEL complex. In a general sense, the primary interface residues are the same: a hydrophobic patch at the centre of the interface composed of Y33, V36 and I38, surrounded by an inter-molecular hydrogen bond network and electrostatic interactions (Figure 5a). In contrast to the NL8-HEL interface, the AM3L09-HEL interface shows an increased number of polar interactions, (going from 10 to 15 inter-molecular hydrogen bonds) and the addition of two salt bridges. Residues W90 and D91, located in L4 of AM3L09, together account for three hydrogen bonds, a salt bridge and burial of 195 Å^2^ at the interface. A very clear rigid-body shift relative to the NL8 binding position is also maintained in AM3L09 (Figure 5d).

In AM3L15 (K_D_ = 35 nM) L4 has adopted a helical character, with no similarity to L4 in AM2EP06. The N-terminus of the helix in L4 is capped by a tryptophan cis-prolyl peptide bond (W86, P87). Intriguingly, this type of bond has been highly conserved across the proteins in which it has appeared during evolution (across all families of protein fold), underscoring its vital functional role [28]. In this case P87 appears to make hydrophobic contacts with HEL, while W86 mediates an inter-molecular hydrogen bond. Two L4 residues (D91 and T92), although not mutated in this library or previously directly involved in binding HEL were recruited for close involvement at the binding interface. In a position similar to Q87 in AM2EP06, the sidechain of T92 makes polar contacts with OBody interface residues of the β-sheet face, while neighbouring residue D91 introduces an inter-molecular salt bridge with HEL. These new contacts are the product of L4 rearrangement brought about by this affinity maturation step.

In both AM3L09 and AM3L15, complementary electrostatic charges are concentrated in two major patches. One consists solely of R35, which associates directly with the negatively charged active site of HEL. The other involves D91, which despiteL4 adopting completely different conformations in these two variants, occupies the same position in both structures, becoming one of three acidic residues (E83, E95 and D91) arranged in a line across the top of the interface, creating a negatively charged patch which binds to a lysine/arginine pair from HEL (Figure 5b and c).

In addition to the protein atoms which interact directly, the AM3L09-HEL interface has a complement of highly-ordered water molecules which mediate both intra- and inter-domain interactions. In particular, two clusters of ordered solvent are closely involved with interface residues, with one anchoring the C-terminal end of L4, and another larger cluster arranged between critical binding residues Y33 and R35 (Figure 5e). Comparison with the AM3L15 and AM2EP06 structures show analogous interactions across the interface in very similar positions at L4, but mediated by protein atoms in both cases. Overall, the improvement in affinity, from 250 nM in AM2EP06, to 35 nM in AM3L15 and finally 3 nM in AM3L09 is correlated with the stabilisation and recruitment of L4 into progressively more substantive contact with HEL. This observation is supported by changes in relative crystallographic B-factors: L4 residues from AM2EP06, AM1L10 and NL8 are generally above the average B-factor or disordered, consistent with a relatively unrestrained surface loop. In contrast, L4 residues from AM3L15 and L09 are at or below the average B-factor, which is more consistent with core or interface residues (Figure 4).

### Thermal Stability of HEL-binding Obodies

**Figure 6:**
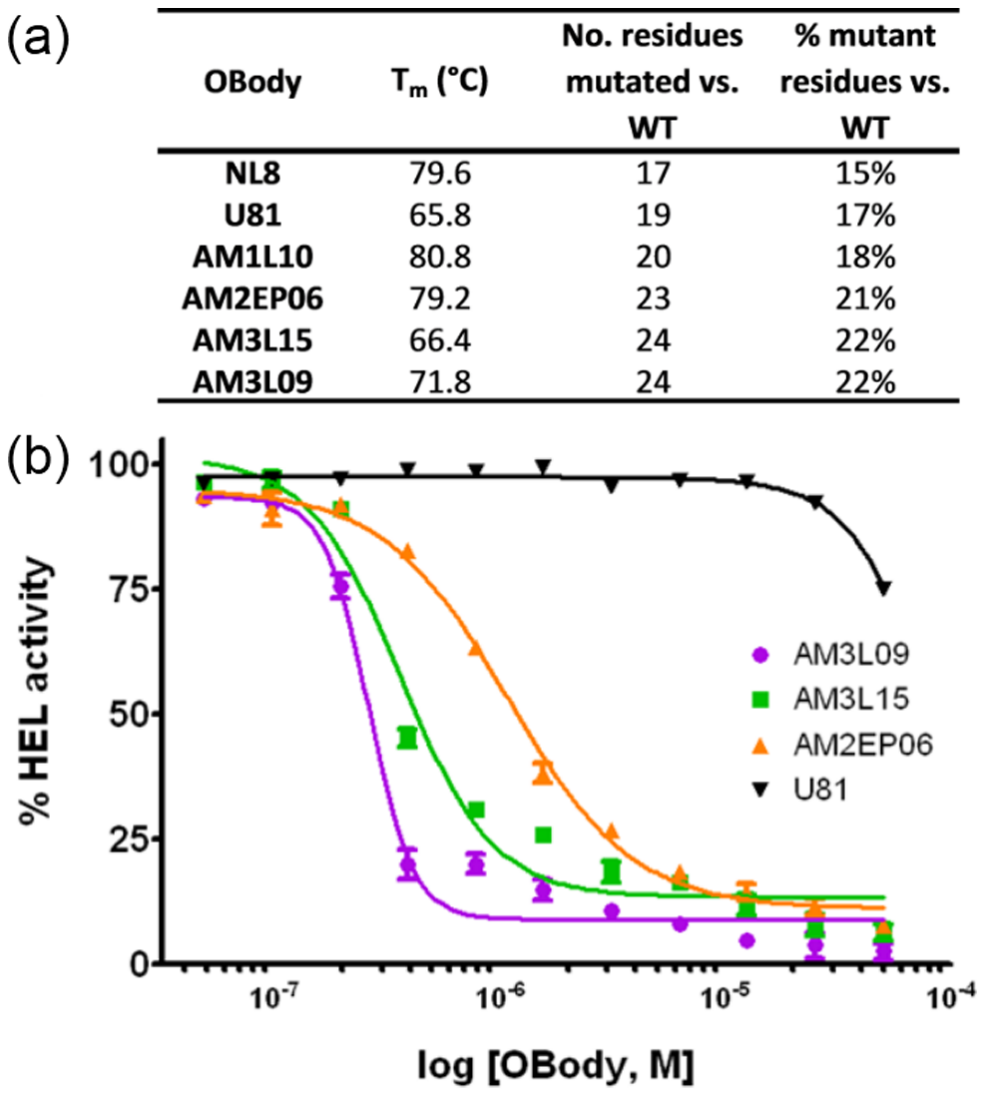
Differential scanning calorimetry thermal denaturation and HEL inhibition assay for HEL-binding and control OBodies. (a) Thermal denaturation by differential scanning fluorimetry of HEL-binding OBodies and a control non-HEL-binding OBody U81. Calculated T_m_ values are shown alongside the number of amino acid mutations as compared to the wild-type AspRS OB-fold domain from *P. aerophilum*. (b) HEL activity assay showing inhibition by OBodies AM3L09, AM3L15 and AM2EP06 and negative control U81. Lines show the nonlinear fit of a variable-slope dose-response model. Error bars show the 95% confidence interval derived from triplicate data points. The data shown are representative of that obtained from multiple independent repeats of the same assay.

Differential scanning fluorimetry [29] was used to estimate the melting temperature (T_m_) of a range of HEL-binding OBodies and a control non-HEL-binding OBody (Figure 6a). These data show that although stability has reduced slightly as the number of mutations increase, the thermophilic character has been maintained despite mutation of up to 22% of the protein.

### Inhibition of HEL Enzymatic Activity

HEL was originally chosen simply as a model protein for selection of OBody binders. However, the similarity of the binding to known HEL inhibitors with respect to interface statistics (Table 2) as well as mode of binding suggested that, with sufficient affinity, this lineage of OBodies had the potential to inhibit HEL enzyme activity. The capacity of AM2EP06, AM3L15 and AM3L09 to inhibit HEL activity was therefore measured by titration against a constant concentration of HEL in the presence of *Micrococcus lysodeikticus* cells to determine the IC_50_. The ranking observed in IC_50_ mirrors that seen for affinity: in the presence of 162 nM HEL, AM3L09 had an IC_50_ of 261 nM, AM3L15 362 nM and AM2EP06 1.1 µM (Figure 6b). The negative control was an OBody with no HEL-binding activity (U81), which showed no effect on HEL activity over the same concentration ranges. Thus, as expected based on the X-ray structural data, the OBodies were able to inhibit specifically HEL enzymatic activity.

**Table 2:** Interface statistics of OBody HEL-binding compared to other known HEL-binding proteins, calculated by PDBePISA.

Molecule	Buried solvent-accessible area (A^2^)	H-bonds	Salt bridges	K_D_
NL8	821	10	5	35 µM
AM1L10	834	10	4	5 µM
AM2EP06	945	10	3	250 nM
AM3L15	974	14	6	30 nM
AM3L09	923	16	6	3 nM
Fab′ (1FDL)	680	14	0	22 nM
Camelid VHH (1JTP)	772	8	0	50 nM
YkfE (1GPQ)	768	15	3	1 nM

## Discussion

The development of OBodies has followed a similar path to other engineered non-antibody scaffolds, with the initial library design and selection producing binders of moderate affinity, in the micromolar range [11], [30]. The subsequent improvements in affinity achieved through phage selection of combinatorial libraries have shown that the OBody scaffold is capable of monomeric affinities in the low nanomolar range, and therefore worthy of further development for specific applications. But, uniquely amongst other engineered non-antibody scaffolds, the progressive affinity maturation process undergone by the HEL-binding OBodies has been thoroughly documented by atomic-resolution X-ray crystallographic structures of representative OBodies in complex with HEL.

Through examination of these structures, we were able to propose a rationale for the effect of the mutations selected at each step, from fine- to broad-scale changes. Selections from the naïve library isolated OBody NL8 (K_D_ = 35 µM); the crystal structure of the NL8-HEL complex at 2.75 Å showed an OBody bound to the active site and substrate-binding groove of HEL, with a central hydrophobic patch made up of Y33, V36 and I38, surrounding polar contacts (11 hydrogen bonds) and three inter-domain complementary electrostatic charges. This structure yielded two critical pieces of information. Firstly, binding to HEL was mediated by residues targeted in the library, showing that the library design was successful, even though it was based on a scaffold for which there was no available structural data. This is unusual for a new scaffold, as crystallographic structural data is usually considered vital as a starting point. The NL8-HEL interface made no apparent use of the four randomised positions in L4 of NL8 (out of a total of 17 distributed throughout the domain), which immediately suggested that the recruitment of L4 into binding interactions might be a viable strategy for affinity maturation. Secondly, the structure of the complex showed that the beta-sheet binding face had statistics which were not excessively different from other HEL-binding proteins, most notably antibodies, strongly suggesting that with appropriate maturation steps the affinity could be improved.

Selections from the first affinity maturation library yielded the NL8 variants AM1L10 (K_D_ = 5 µM) and AM1L200 (K_D_ = 1 µM), representing 6- and 35-fold improvements in affinity over NL8, respectively. The crystal structure of the AM1L10-HEL complex at 1.95 Å revealed two major factors in the improvement in binding: the introduction of additional inter-domain contacts at Y56, and a consequent moderate shift in binding orientation compared to the NL8-HEL complex. To some extent the pattern-recognition function of hydrogen bond networks is similar in both intra- and inter-molecular interactions [31]. Where they differ is in the degree to which they can be optimised. Norel and co-workers [32], [33] showed that geometric complementarity can be used successfully as the sole docking criteria for finding binding sites, even when using structures of monomers not solved in the presence of their ligand. This strongly implies that, at least in the cases examined, the binding surface shape is largely determined before binding, essentially restricting unsatisfied hydrogen bonds on the interface of a folded structure to a rigid-body search. Consequently, bonds formed between proteins present a much broader range of angles and distances than found in other contexts [34], [35], impacting negatively on bond stability as they diverge from the theoretical ideal. There are cases where large conformational shifts can be seen on binding, most obviously in domain-swapping dimers or natively unfolded proteins (e.g. βγ-crystallin on calcium binding [36]) but it has been noted that antibody-antigen complexes exhibit comparatively little conformational changes as a result of binding [37], and that amongst the available degrees of freedom for a particular residue, a strongly-binding but infrequently-sampled conformational isomer contributes poorly to the free energy of binding [38], [39]. Thus, the shift in binding orientation seen in AM1L10, and later in AM2EP06, can be viewed as an overall optimisation of bonding across the entire binding face, with the OBody “settling” on to the HEL ligand.

For the second phase of affinity maturation, AM1L200 was used as the template for an EP-PCR library, selections from which produced variant AM2EP06 (K_D_ = 250 nM), representing a 4-fold improvement in affinity over AM1L200. The crystal structure of the AM2EP06-HEL complex was solved at 1.86 Å. In this case the exact molecular determinants of the improvement in affinity were less clear, but the most likely explanation seemed to be a change in L4 dynamics through the K86E substitution. Residue E86 from AM2EP06 made limited contacts with HEL, and its sidechain was poorly-ordered. Although some role in long-range electrostatic attraction may be hypothesised, we think it most likely that this mutation increased the occupation of binding-favourable conformations of L4 by removing competing interactions with HEL. Indeed, the NL8 and AM1L10 structures both showed K86 in a HEL-contacting position which could not be accommodated at the same time as the L4 arrangement following the K86E substitution in AM2EP06.

In the third and final phase of affinity maturation, six residues in L4 of AM2EP06 were randomised and selections from this library resulted in the isolation of AM3L15 (K_D_ = 30 nM) and AM3L09 (K_D_ = 3 nM), constituting 8- and 83-fold improvements in affinity over AM2EP06, respectively. In this case, selections were performed in solution using biotinylated target and trypsin-sensitive helper phage KM13 [40], which we expect greatly increased their efficiency over selections performed with previous libraries (given that the latter were done with solid-phase selections and VCSM13 helper phage). The crystal structures of the AM3L15-HEL and AM3L09-HEL complexes were solved at 1.86 Å and 2.57 Å, respectively. These two final structures showed unequivocally that the improvements in affinity were due to recruitment of conformationally-restrained L4 residues into direct contact with HEL (Figure 4). They also demonstrated that the core OBody fold is capable of accommodating very different loop sequences, including a cis-prolyl bond capping a short α-helix in the case of AM3L15, as well as the extended conformations seen in AM2EP06 and AM3L09. With HEL-binding OBodies in the low nM-range, inhibition of HEL activity could be shown, both demonstrating a model application for engineered OBodies and providing further confirmation of binding independent of SPR-based measurements.

While the naïve library selections were clearly successful, indicating that the library design was sufficient, the structures solved subsequently showed an important property of the designed interface; interactions with HEL relied heavily on, and were consequently limited by, the involvement of wild-type residues. This is, at least partially, a consequence of the way NL8 bound its first β-strand into the HEL substrate binding groove, which forced non-randomised residues on the underside of the β-strand to make contact with HEL residues. In addition, the two regions targeted, namely L4 and the β-sheet binding face, did not form a continuous surface, which resulted in non-randomised residues from β-strands 4 and 5 also being forced into contact. Both of these non-randomised regions contain charged residues; especially important is E95 on β-strand 5, positioned directly between L4 and the β-sheet interface. While this may or may not be an issue for binding epitopes on a given target, we may be able use this most recent structural information to design an improved binding surface, by repositioning the scaffold binding region and removing charged groups which might otherwise interfere.

In addition, these five structures provide an empirical basis for design of OBody libraries in the future, enabling precise selection of target residues for mutations and assessment of resulting hypothetical binding surfaces without the need for further low-throughput structural studies of the kind employed here.

OBodies can also be produced easily by expression in *E. coli* and without performing any optimisation we have been able to achieve purified yields of 100–200 mg per litre of culture.

We have shown that an OBody scaffold is capable of binding in monomeric form with nanomolar affinity to HEL, a model target ligand. Detailed structural information at each step, from naïve clone and through the affinity maturation process showed an iterative improvement in interface statistics, and demonstrated the scaffold’s ability to maintain highly diverse sequences (mutating up to 22% of the protein) with no appreciable change in core residue arrangement (Cα r.m.s.d. across all of the structures of 0.2 Å, excluding surface loops ) and maintenance of the thermophilic character of the ancestral OB-fold domain (T_m_ 72°C for AM3L09). Due to their small size (13-fold smaller than IgGs), lack of disulfides and superior biophysical characteristics, OBodies derived from thermophilic bacterial OB-fold domains may be useful for extended-shelf-life *in vitro* diagnostics or as tools for detection or bioseparation. Further applications in therapeutics may yet be possible, either with OBodies derived from human OB-folds or engineered de-immunised versions of the bacterial OBody scaffold presented here.

## Materials and Methods

### Naïve and First Affinity Maturation Library Construction

These libraries were constructed by PCR dissection of the ancestor gene into overlapping fragments followed by overlap-extension assembly incorporating degenerate oligonucleotides. The naïve OBody library based on the OB-fold from *Pyrobaculum aerophilum* aspartyl tRNA synthetase (GenBank ID NP_558783.1; residues 1–109) was constructed in the pRpsp2 phagemid [23] using PCR and overlapping oligonucleotides containing NNK (N = G, A, T or C; K = G or T) codons, randomising the amino acids at 17 positions across the domain and introducing a 6 bp insertion immediately following wild-type residue 46 which encoded the residues GA, to facilitate library construction (Figure 1b and Figure S5). For the naïve library, mutagenic oligos 051, 052 and 053 were used (see Table S1 for oligo sequences and Figure S4 for detailed construction plan). The first affinity maturation library was made using PCR and overlapping oligonucleotides containing NNK codons and a single NRK (R = G or A) codon in pRpsp2. For this library, mutagenic oligos 183, 184 and 163 were used (see Table S1 for oligo sequences and Figure S5 for detailed construction plan). The intact gene libraries were digested with *Nco* I and *Not* I and ligated into phagemid pRpsp2 using a T4 ligase reaction with an insert:vector ratio of 3∶1 (9 pmol vector, 3 pmol library insert). DNA was purified from the ligation by ethanol precipitation to reduce the volume, then on a column using a Roche HiPure PCR Purification kit. The ligated DNA was transformed into freshly-prepared electrocompetent TG1 *E. coli* in 50 µL aliquots. Library size was estimated by serial dilution and colony count.

### Second Affinity Maturation Library Construction

Error-prone (EP)-PCR [41] was used to generate a randomly-mutated library. EP-PCR was performed using *Taq* DNA polymerase and primer pair 005 and 006 (Table S1). Polymerase errors were induced by the introduction of 0.5 mM MnCl_2_ to reduce polymerase specificity. Also, deoxynucleotide-triphosphates thymidine and cytidine were disproportionately increased, from 0.2 mM to 1 mM, to increase the likelihood of misincorporation. MgCl_2_ was increased to 7 mM to reduce primer specificity. To reduce the chances of non-specific products, the template gene was introduced as a freshly-prepared PCR product, at 10 fmol per 100 µL reaction. Taq polymerase concentration was also increased, to 5 U per reaction. The mixture was split into 10 µL aliquots for cycling to prevent dominance of an early mutation in the library as a whole. The EP-PCR product was gel-purified and the whole library amplified in a normal PCR reaction with phagemid cloning primers 192 and 040 (Table S1) to produce sufficient quantities for cloning. The final PCR product library was gel-purified and digested with *Nco* I and *Not* I for cloning into the pRpsp2 phagemid.

### Third Affinity Maturation Library Construction

The third affinity maturation library was made using Kunkel mutagenesis with NNS randomisation in the pAS1 phagemid, essentially as described elsewhere [42]. Single-stranded template DNA containing uracil (dU-ssDNA) was generated from phagemid pAS1-AM2EP06, following growth in and isolation from, *E. coli* CJ236. A single colony was used to inoculate 200 mL LB, supplemented with 0.25 µg/mL uridine and 100 µg/mL carbenicillin, and grown at 250 rpm, 37°C until OD_600_ = 0.1–0.2. Helper phage KM13 [40] was added to the culture at a multiplicity of infection of 20 and the culture incubated at 250 rpm, 37°C for 1 h. Kanamycin was added to 50 ug/mL and the culture incubated overnight at 250 rpm, 37°C. The cells were removed by centrifugation and discarded. Phage were precipitated from the supernatant by the addition of PEG 8,000 to 4% (w/v) and NaCl to 3% (w/v) and incubation on ice for 1 h. The precipitate was harvested by centrifugation at 15,000×*g* for 15 min, the supernatant discarded and the pellet dissolved in 4 mL PBS pH 7.4. The final solution was centrifuged at 15,000×*g* for 15 min, then filtered through a 0.45 µm membrane. dU-ssDNA was isolated from phage using a Qiagen M13 preparation kit.

Generation of heteroduplex DNA was performed using degenerate oligonucleotide 247 (Table S1). The oligonucleotide was phosphorylated by treatment with T4 Polynucleotide kinase, annealed to the template dU-ssDNA, then extended and ligated using Klenow fragment and T4 Ligase. The heteroduplex DNA was purified using a Roche High Pure PCR Purification kit and transformed into freshly prepared electrocompetent TG1 *E. coli* in 400 µL aliquots. Library size was estimated by serial dilution and colony counting.

### Solid-phase Phage Selection

Purified phage were generated using VCSM13 helper phage (Agilent Technologies, USA), packaging the pRpsp2 phagemid [23]. Nunc immunotubes (ThermoFisher Scientific, Waltham MA, U.S.A.) were used as the initial, solid-phase method of ligand immobilisation for panning experiments. Ligand in 2 mL PBS was incubated with constant shaking overnight at room temperature in parafilm-sealed immunotubes. Each tube containing ligand was matched to a control tube with an equal concentration of BSA. After the overnight incubation, each tube was blocked for 3 h in 4 mL PBS with 1% BSA (w/v) and rinsed five times with 5 mL PBS. Input phage samples were pre-adsorbed with PBS with 0.1% BSA for at least 1 h, then panned in 2 mL aliquots per immunotube for 1 h with gentle stirring. The supernatant was discarded and each tube washed 10 times with 5 mL PBS with 0.1% Tween-20 (PBS-T), then five times with 5 mL PBS. Bound phage were eluted with 1 mg/mL trypsin in PBS with 1 mM CaCl_2_ and orbital mixing.

Eluted phage from a single selection were added to 10 mL log-phase TG1 *E. coli* (grown in 2TY) and incubated for 1 h at 37°C, 150 rpm. Phage titre was determined by plating 100-fold serial dilutions of the infected culture on 2TY agar with 100 µg/mL carbenicillin and counting the resultant colonies. The remainder of each phage output was reduced in volume to ∼1–2 ml by centrifugation at 4000 g for 10 min before plating on a bioassay dish (Nunc) containing the same medium and incubation at 30°C overnight. For the second and subsequent rounds of selection, 100 µL of filtered (0.45 µm) culture supernatant was used as the input phage sample. To determine the input titre, 10 µL of the supernatant was diluted 10,000-fold in PBS and used to infect 990 µL log-phase TG1 for 1 h at 37°C, 150 rpm, then plated and incubated as above.

### Solution-phase Phage Selection

Purified phage were generated using KM13 helper phage [40], packaging the pRpsp2 phagemid [23]. For a single selection experiment, 100 µL of Dynal M280 streptavidin magnetic beads (Life Technologies) were washed three times with 1 mL PBS and blocked for 1 h with 1 mL PBS-T with 3% (w/v) skim milk powder (PBS-TM) on a rotator at room temperature. The input phage were deselected in PBS-TM with 50 µL blocked streptavidin beads by incubation for 1 h on a rotator at room temperature. Streptavidin beads were removed from the input phage and biotinylated ligand was added to a final concentration of 1 µM for the first-round selections, 10 nM for the second-round selections and 1 nM for the third-round of selections. Solution-phase binding was allowed to proceed for 2 h on a rotator at room temperature. To recover ligand-bound phage complexes, a 50 µL aliquot of blocked streptavidin beads at 10 mg/ml was added to the selection for 10 min on a rotator at room temperature. The beads were then collected and washed eight times with 1 mL PBS-T. Bound phage were eluted by the addition of 1 mL PBS with 1 mM CaCl_2_ and 1 mg/mL Trypsin and incubation for 1 h at 37°C with orbital mixing. Eluted phage were infected and titrated as indicated above for solid-phase selections.

### Phage ELISA

To generate monoclonal phage samples and perform phage ELISAs for screening selections from the third round of affinity maturation, individual clones were grown in 100 µL 2YT with 100 µg/mL carbenicillin in a 96-well tissue culture plate overnight at 37°C, 150 rpm. The resulting saturated cultures were seeded into 500 µL 2YT with 100 µg/mL carbenicillin in 96-deepwell (2 mL) blocks and incubated for approximately 5 h at 37°C, 250 rpm, or until turbidity was evident. The cultures were infected with KM13 helper phage at ∼10^9^ pfu and incubated at 37°C, 150 rpm, for 1 h. Media was exchanged by centrifugation for 10 min at 3,000 *g*, carefully discarding the supernatant and resuspension in fresh 2YT with 100 µg/mL carbenicillin and 50 µg/mL kanamycin. Plates were grown overnight at 25°C, 250 rpm. As a negative control, phage displaying the wild-type AspRS OB-fold domain were produced in a separate plate by the same method. To prepare the phage in each well for ELISA, 500 µL of PBS with 6% w/v skim milk powder was added to each well, incubated for 1 h, then the cells pelleted. Nunc Maxisorp 96-well plates were coated with HEL or BSA by incubation with 50 µL at 50 µg/mL overnight at 4°C. Each plate was rinsed 3 times with 300 µL PBS, blocked with 300 µL PBS containing 3% (w/v) skim milk powder (PBS-3M) for 1 h and washed again as above.

From the deepwell plate, 50 µL of the prepared phage solution was transferred in duplicate to the ligand and control plates then incubated at room temperature for 1 h. Anti-HEL human antibody (HuCAL Clone 11397, AbD Serotec, Oxford, UK) 50 µL in PBS-3M at 1 µg/mL was used as a positive control. A blank negative control well with 50 µL PBS-3M only was also used. The wells were washed three times with 300 µL PBS-T, then probed for 1 h with 50 µL monoclonal HRP-conjugated anti-M13 antibody (dilution 1∶5000, GE Healthcare), or HRP-conjugated polyclonal goat anti-human Fc (dilution 1∶1000, AbD Serotec) in the case of the positive control. The plates were washed 3 times as above. For visualisation, 50 µL 3,3′,5,5′-tetramethylbenzidine (TMB) peroxidase substrate (Sigma) per well was added and incubated for 30 min at room temperature, then the reaction stopped with 50 µL 2 M H_2_SO_4_ per well and read in in a spectrophotometer at 450 nM.

To generate monoclonal phage samples and perform phage ELISAs for screening all other selections, 96-well ELISA plates were coated with 5 µg/ml hen egg white lysozyme or 1% BSA, in PBS at 4°C overnight. After two washes with PBS, plates were blocked with PBS containing 5% (w/v) skim milk powder (PBS-5M) for 1 h at RT before helper phage (10^9^ per well; ΔgIII helper phage VCSM13d3 [43]) were added in PBS-T containing 2.5% (w/v) skim milk powder. Plates were incubated for 2 hours at RT with agitation. After 10 washes with PBS, monoclonal HRP-conjugated anti-M13 antibody (GE Healthcare) diluted in PBS-5M was added and incubated for 1 h at RT. Plates were washed 4 times with water and HRP-conjugated rabbit-anti-mouse antibody (Pierce) in PBS-5M was added to the wells and incubated for 1 h at RT. Plates were washed 4 times with water and 50 µl substrate solution (1 mg/ml o-phenylene-diamine in PBS and 0.03% H_2_O_2_) added per well. The reaction was stopped after ∼15 min by addition of 25 µl 2.5 M H_2_SO_4_ and the absorbance was recorded at 492 nm.

### Differential Scanning Fluorimetry

Measurement of changes in protein stability was performed using the general method for fluorescent detection with SYPRO Orange and an RT-PCR instrument, outlined elsewhere [29]. Briefly, protein samples were melted using a Corbett Rotogene 6000 RT-PCR machine, in duplicate at both 150 and 300 µg/mL, with SYPRO Orange at 100x concentration in a total volume of 25 µL per sample. The temperature was ramped at 1°C per minute, from 25°C to 99°C, with excitation at 470 nM and emission at 555 nM. Curve fitting to obtain the T_m_ was performed with GraphPad Prism, using the following equation, where yMin and yMax are the minimum and maximum fluorescence values, h is Hill’s slope and T_m_ is the point of inflection:
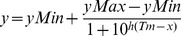



### Affinity Measurements

Dissociation constants of the various Obodies for HEL were measured using a Biacore 3000 surface plasmon resonance instrument (GE Healthcare). HEL was immobilised on to a CM5 sensor chip in small steps to give approximately 100 response units (RU). Obodies were measured in 2-fold dilution series, beginning with at least 10-times the K_D_, where known. Analysis runs were performed at a standard 50 µL/min using HBS-EP running buffer (GE Healthcare). Chip surfaces were regenerated after each analyte injection with two 10 µL injections of 1 M NaCl. Where possible, kinetic data was modelled to obtain the k_on_/k_off_ using global fitting to a 1∶1 Langmuir binding model in BIAevaluation software version 3.2, otherwise steady-state dissociation constants were modelled by plotting the maximum response at each concentration vs. OBody concentration in mol/L and fitting a Langmuir saturation binding curve to the data, using GraphPad Prism with the following equation, where Rmax is the maximum response, K_D_ is the dissociation constant and for the linear component, m is the gradient and c is the y-intercept of the linear portion:
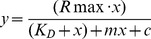



### Inhibition of HEL Enzymatic Activity

Assays were carried out in triplicate, using flat-bottomed 96-well untreated ELISA plates (Greiner). OBodies suspended in PBS pH 7.4 were first diluted 1∶2 serially over 11 steps, starting with a final assay concentration of 50 µM. A 25 µL aliquot of each OBody dilution was either added to 25 µL hen egg-white lysozyme (Sigma) at 648 nM (162 nM final concentration in assay) or 25 µL PBS pH 7.4 and allowed to equilibrate at room temperature for 15 min. To each OBody dilution, a 50 µL aliquot of substrate solution containing inactivated *Micrococcus lysodeikticus* cells (Sigma, M3770) suspended in PBS pH 7.4 was added to a final concentration of 0.4 mg/mL and plates were incubated at room temperature for 45 min before reading the absorbance at 450 nm in a BMG Fluostar Optima plate reader. The data were normalised and processed using Prism v5.04 (Graphpad) software and non-linear regression performed using the following model [Y = Bottom+(Top-Bottom)/(1+10<$>\vskip -3 \raster(60%)="rg1"<$>((LogIC_50_−X)*HillSlope))] to obtain IC_50_ values.

### X-ray Crystallography

All crystal were grown using the sitting-drop vapour-diffusion method. Conditions were as follows: NL8, 0.2 M HEPES pH 7.3, 7% MPEG 5,000; AM1L10, 0.2 M HEPES pH 7.4, 9% MPEG 5,000; AM2EP06, 0.2 M HEPES pH 7.0, 13% MPEG 5,000; AM3L15, 0.2 M HEPES pH 7.4, 5% MPEG 5,000; AM3L09, 0.2 M HEPES pH 7.8, 9% MPEG 5,000. Purified, concentrated OBody in PBS pH 7.4 was combined with equimolar HEL in 10 mM sodium acetate pH 5.0 to yield concentrations of 40 mg/mL (OBody) and 45 mg/mL (HEL). Diffraction data was collected using the home source at the Maurice Wilkins Centre, University of Auckland, the Stanford Synchrotron Radiation Lightsource, or the Australian Synchrotron MX2 beamline. Data were integrated using MOSFLM [44], reduced using SCALA [45]/TRUNCATE [46], phased by molecular replacement with PHASER [47], refined using CCP4 [48], PHENIX [49] and COOT [50] and visualised with PyMOL [51]. TLS parameters for refinement were determined using the TLSMD server [52], [53].

### Biotinylation of Hen Egg-white Lysozyme

Biotinylated HEL was produced using an amine-reactive biotinylation reagent from Qanta Biodesign, which attaches a biotin with a polyethylene linker via an N-hydroxysuccinimide group. The reagent was dissolved in dry dimethyl sulfoxide (DMSO) to make a 100 mM stock solution and stored at −20°C. The HEL was obtained from Roche and purified on a Superose 75 10/300 size exclusion column and incubated with a 5-fold molar excess of the biotinylation reagent for 1 h. The reaction was halted with the addition of ethanolamine to 1 mM. The sample was dialysed into ultrapure water and analysed for biotinylation levels by MALDI-TOF mass spectrometry.

### Protein Purification

Selected OBodies were subcloned by restriction/ligation into expression vector pRoEx Htb (Life Technologies) using generic OBody primers 005 and 006 (Table S1). Cultures for expression were inoculated from seeder cultures into baffled flasks of Luria Broth (LB) with 100 µg/mL carbenicllin and grown at 37°C. Induction of expression in host *E. coli* strain DH5α was at an OD_600_ of 0.5–0.8 with the addition of IPTG to 1 mM, overnight at 37°C. OBodies were purified as follows: cell lysis by sonication in PBS, solution fraction separated by centrifugation at 15,000 *g* for 20 min, followed by nickel IMAC with a 5 mL HisTrap FF column (GE Healthcare), removal of the 6xHis tag by digestion with recombinant tobacco etch virus protease and size exclusion purification with a Superose 75 10/300 analytical-grade column (GE Healthcare).

### Accession Numbers

Coordinates and structure factors for the reported crystal structures have been deposited at the Protein Data Bank under accession codes 4GLA (NL8-HEL), 4GN3 (AM1L10-HEL), 4GN4 (AM2EP06-HEL), 4GLV (AM3L09-HEL) and 4GN5 (AM3L15-HEL).

## Supporting Information

Figure S1 **SPR equilibrium analysis of naïve HEL-binding OBodies.** Affinities were calculated using Graphpad Prism software and an equilibrium model of maximum response, as described in Materials and Methods.
(TIF)

Figure S2 **SPR equilibrium analysis of HEL-binding OBodies from AM1 selections.** Affinities were calculated using Graphpad Prism software and an equilibrium model of maximum response, as described in Materials and Methods.
(TIF)

Figure S3 **AM2EP06-HEL complex structure detail, showing AM2EP06 L4-HEL contacts.** Viewed from within the bound HEL (green Cα trace), L4 contacts with E95 at the top of the AM2EP06 (pale blue Cα trace) β-sheet interface are shown. Dashed yellow lines are potential hydrogen or electrostatic bonds, labelled with distances in angstroms.
(TIF)

Figure S4 **Sequence plan showing detailed construction method for the naïve library.** Blue arrows represent mutagenic oligonucleotides. Green arrows represent non-mutagenic oligonucleotides used for amplifying individual fragments and performing final gene reconstruction. Refer to Supporting Information Table S1 for oligonucleotide sequences.
(TIF)

Figure S5 **Sequence plan showing detailed construction method for the first affinity-maturation library.** Blue arrows represent mutagenic oligonucleotides. Green arrows represent non-mutagenic oligonucleotides used for amplifying individual fragments and performing final gene reconstruction. Refer to Table S1 for oligonucleotide sequences.
(TIF)

Table S1 **Oligonucleotide sequences.**
(XLSX)
