# Supplementary figures and images for: Tracking Molecular Recognition at the Atomic Level with a New Protein Scaffold Based on the OB-Fold
Source: PLoS One. 2014 Jan 20;9(1):e86050. doi: 10.1371/journal.pone.0086050 (PMC3896448; doi:10.1371/journal.pone.0086050)

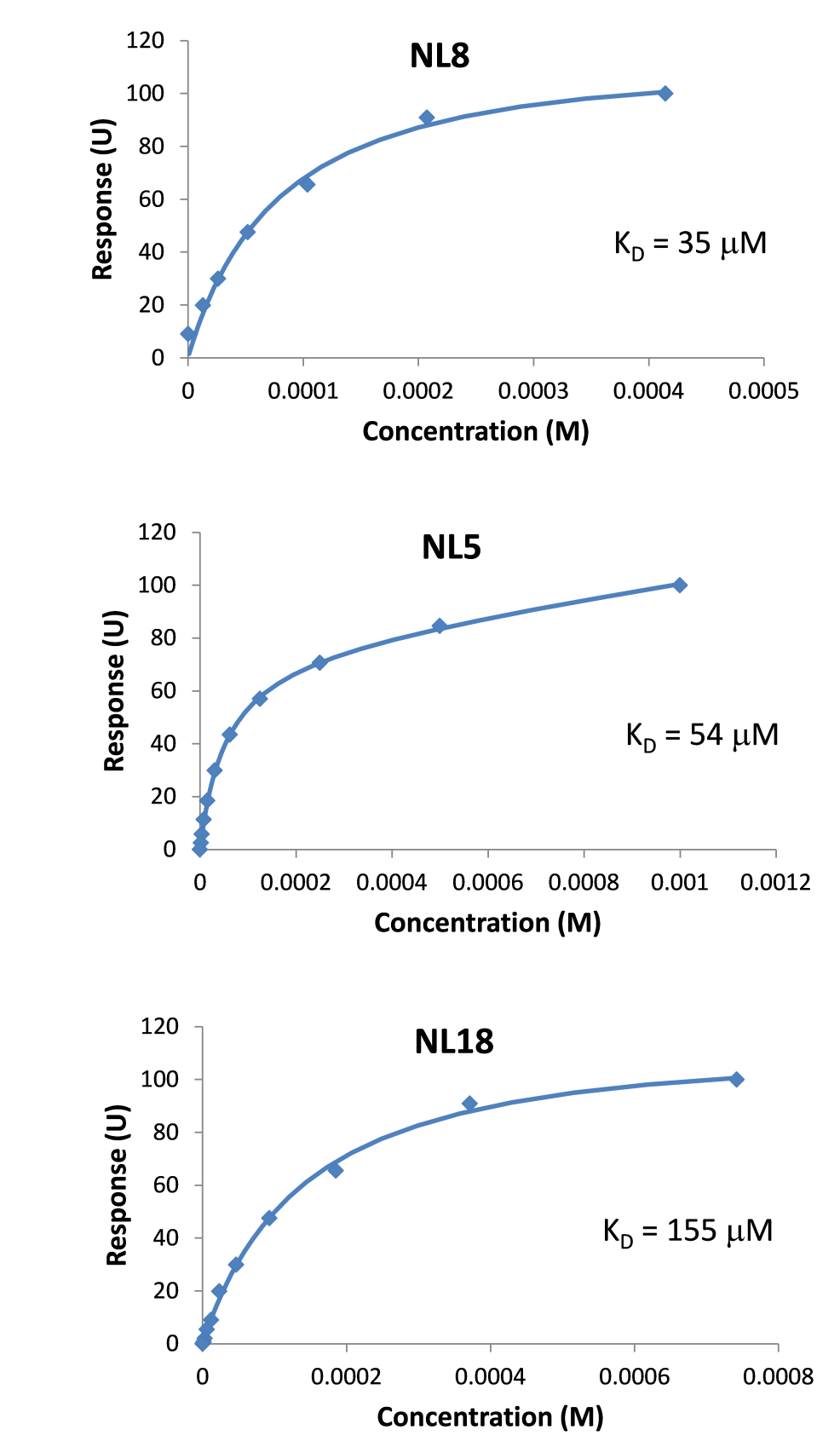

Supplement: Figure S1 — SPR equilibrium analysis of naïve HEL-binding OBodies. Affinities were calculated using Graphpad Prism software and an equilibrium model of maximum response, as described in Materials and Methods. (TIF) [file pone.0086050.s001.tif]

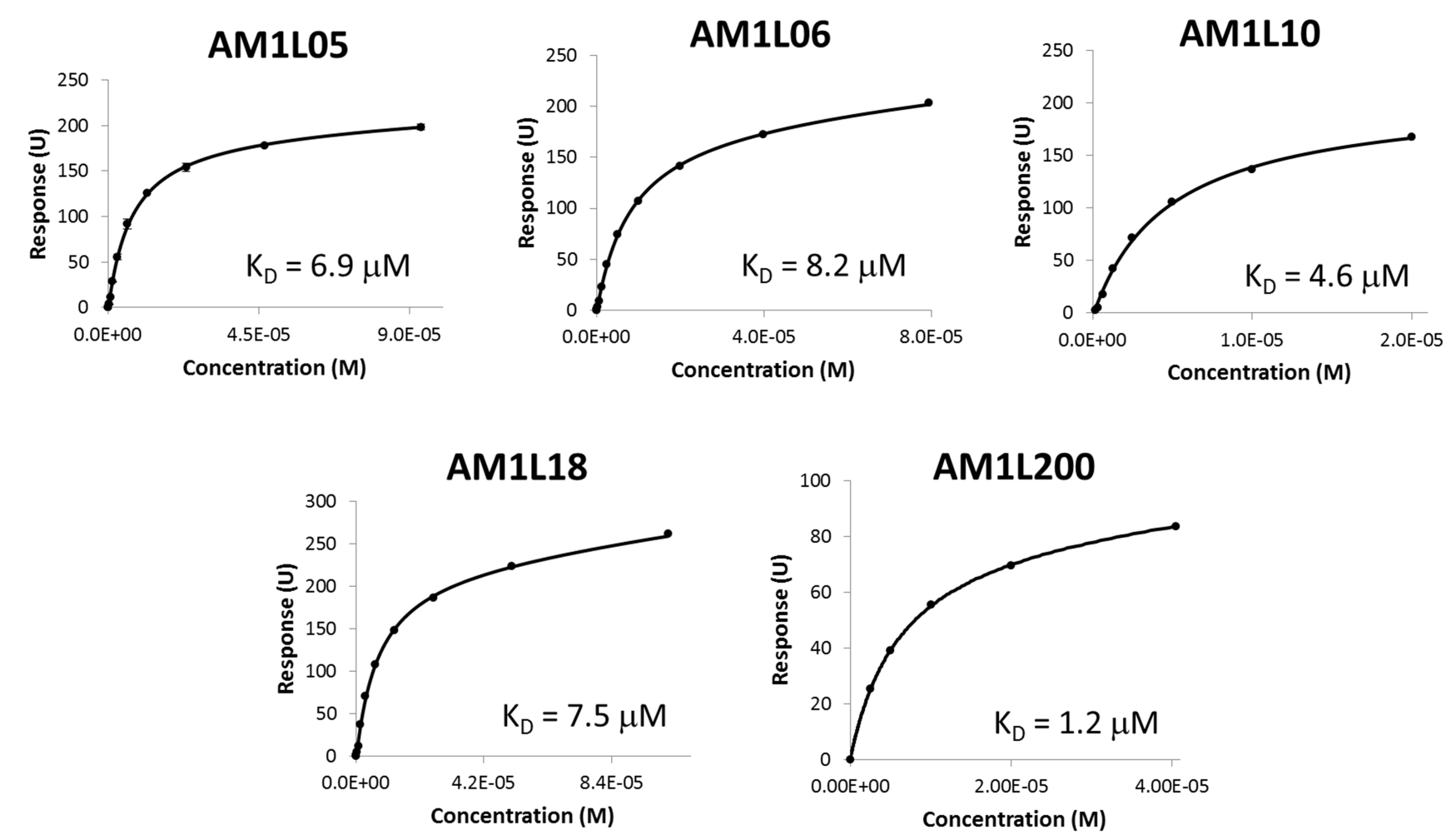

Supplement: Figure S2 — SPR equilibrium analysis of HEL-binding OBodies from AM1 selections. Affinities were calculated using Graphpad Prism software and an equilibrium model of maximum response, as described in Materials and Methods. (TIF) [file pone.0086050.s002.tif]

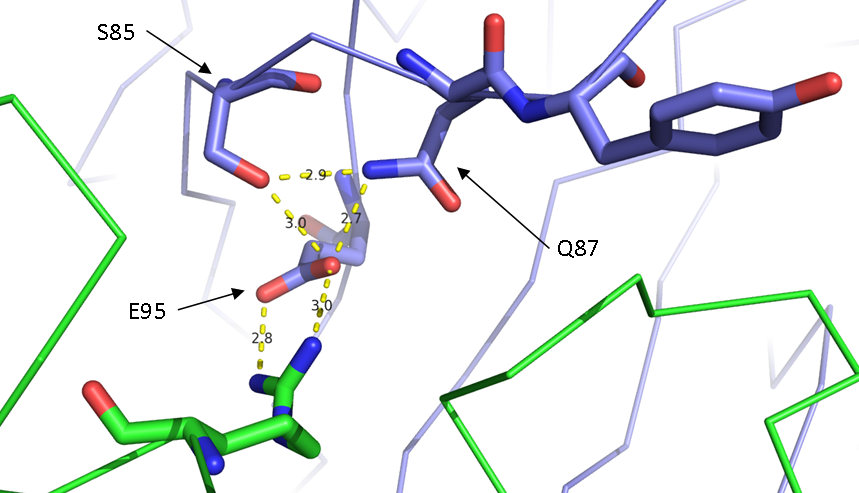

Supplement: Figure S3 — AM2EP06-HEL complex structure detail, showing AM2EP06 L4-HEL contacts. Viewed from within the bound HEL (green Cα trace), L4 contacts with E95 at the top of the AM2EP06 (pale blue Cα trace) β-sheet interface are shown. Dashed yellow lines are potential hydrogen or electrostatic bonds, labelled with distances in angstroms. (TIF) [file pone.0086050.s003.tif]

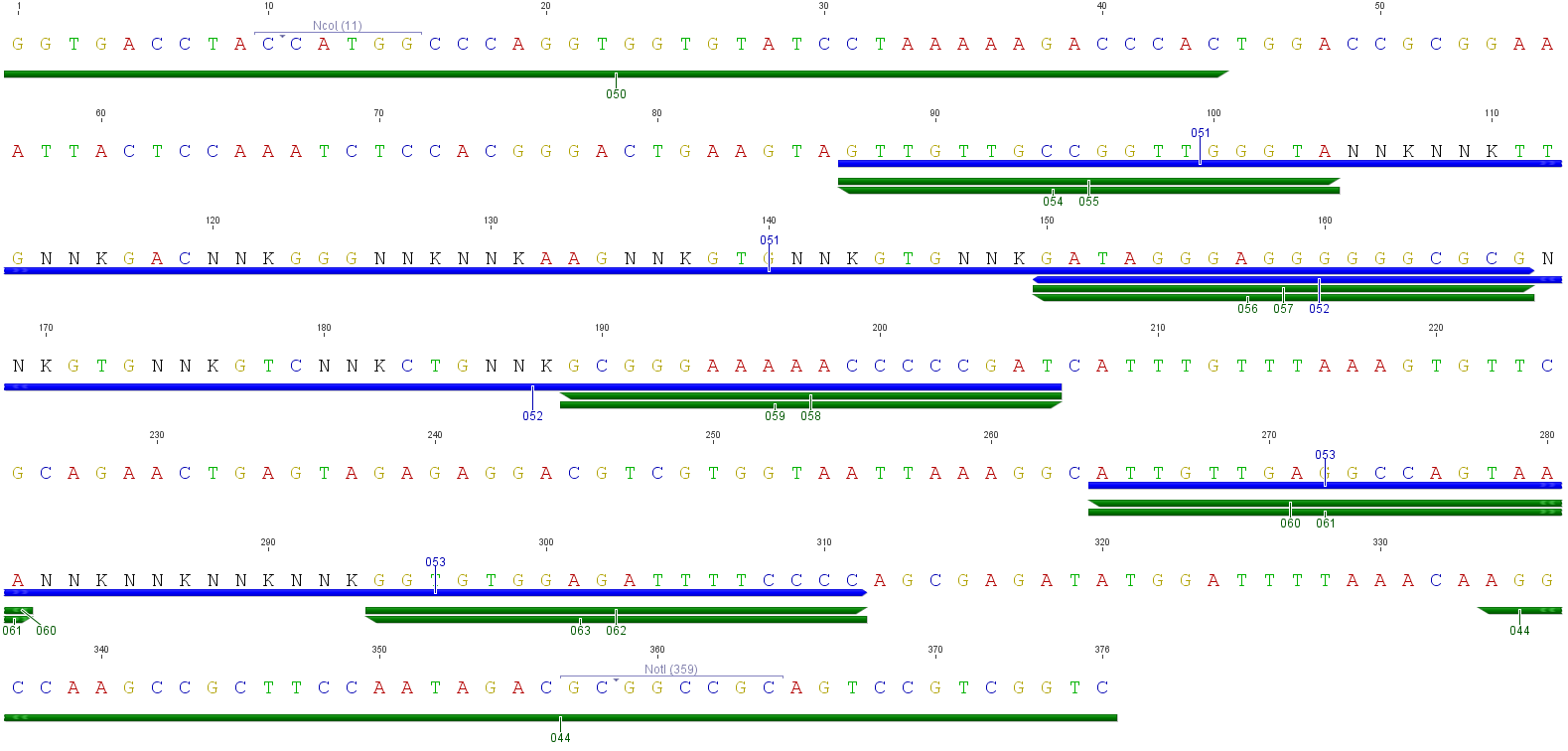

Supplement: Figure S4 — Sequence plan showing detailed construction method for the naïve library. Blue arrows represent mutagenic oligonucleotides. Green arrows represent non-mutagenic oligonucleotides used for amplifying individual fragments and performing final gene reconstruction. Refer to Supporting Information Table S1 for oligonucleotide sequences. (TIF) [file pone.0086050.s004.tif]

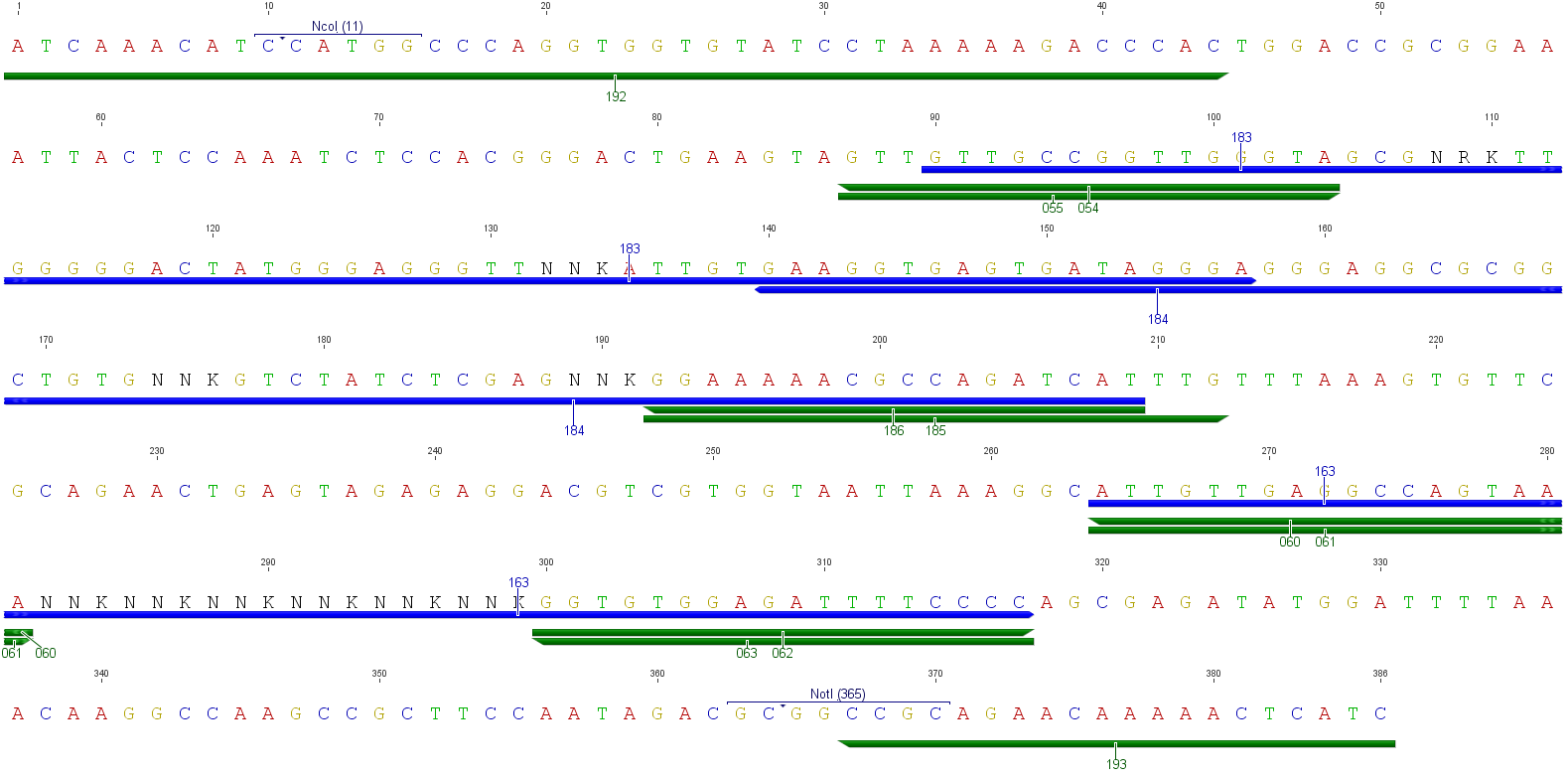

Supplement: Figure S5 — Sequence plan showing detailed construction method for the first affinity-maturation library. Blue arrows represent mutagenic oligonucleotides. Green arrows represent non-mutagenic oligonucleotides used for amplifying individual fragments and performing final gene reconstruction. Refer to Table S1 for oligonucleotide sequences. (TIF) [file pone.0086050.s005.tif]
